# Quartz Enhanced Photoacoustic Spectroscopy Based on a Custom Quartz Tuning Fork

**DOI:** 10.3390/s19061362

**Published:** 2019-03-19

**Authors:** Maxime Duquesnoy, Guillaume Aoust, Jean-Michel Melkonian, Raphaël Lévy, Myriam Raybaut, Antoine Godard

**Affiliations:** 1 Mirsense, Nanno-INNOV, building 863, 8 Avenue de la Vauve, 91120 Palaiseau, France; guillaume.aoust@mirsense.com; 2 DPHY, ONERA, Université Paris Saclay, F-91123 Palaiseau, France; jean-michel.melkonian@onera.fr (J.-M.M.); Raphael.Levy@onera.fr (R.L.); antoine.godard@onera.fr (A.G.)

**Keywords:** QEPAS, photoacoustic spectroscopy, quartz tuning fork

## Abstract

We have designed and fabricated a custom quartz tuning fork (QTF) with a reduced fundamental frequency; a larger gap between the prongs; and the best quality factor in air at atmospheric conditions ever reported, to our knowledge. Acoustic microresonators have been added to the QTF in order to enhance the sensor sensitivity. We demonstrate a normalized noise equivalent absorption (*NNEA*) of 3.7 × 10^−9^ W.cm^−1^.Hz^−1/2^ for CO_2_ detection at atmospheric pressure. The influence of the inner diameter and length of the microresonators has been studied, as well as the penetration depth between the QTF’s prongs. We investigated the acoustic isolation of our system and measured the Allan deviation of the sensor.

## 1. Introduction

Detection of hazardous chemicals and greenhouse gases is one among the many challenges of our century. One of the difficulties lies in the fact that the gases that we need to detect are at trace level, from a few percent to a few ppbv (part per billion in volume), with large variations of the concentration in time. The ability to detect several gases with a single detector is a highly desirable feature, but is hard to achieve. In addition, to be deployed in the field, it is convenient to have a detection technique allowing one to keep a good sensitivity while reducing the overall volume and with a low power consumption. A technique meeting all these requirements is photoacoustic spectroscopy—it presents a wide dynamic range and a very good linearity, and enables multi-species detection with a single and compact device. In this technique, a pressure wave is generated by relaxation of the gas after it has been excited by absorbed modulated light through molecular transitions [1].

The sensitivity of a photoacoustic detector can be qualified by a figure of merit, called normalized noise equivalent absorption (*NNEA*). It represents the minimum detectable absorption, independent from the laser power, the electrical bandwidth, and the absorption coefficient of the gas. It is defined as follows:

(1) $$NNEA=\frac{P\times \;\alpha }{SNR\times \sqrt{\Delta f}}$$

where *P* is the average optical power in W; *α* is the target gas absorption in cm^−1^; Δ*f* is the detection bandwidth in Hz; and *SNR* is the signal to noise ratio, taken as the ratio of the signal root mean square (RMS) value with the RMS noise within the same bandwidth.

A new technique called quartz enhanced photoacoustic spectroscopy (QEPAS) was introduced in 2002 [2], providing a good tradeoff between compactness and sensitivity of the acoustic detector, as well as good acoustic isolation from the environment. This technique involves the generation of a pressure wave through gas excitation with an amplitude- or wavelength -modulated laser and its detection with a quartz tuning fork (QTF), usually complemented by longitudinal acoustic resonators.

### 1.1. Commercial Quartz Tuning Forks

The first available QTFs came from the watch industry. They are generally used under vacuum for time reference applications, but also in air for atomic force spectroscopy [3], for example. However, they present two major drawbacks for photoacoustic spectroscopy—a small gap between their prongs (~200–300 μm) and a high resonance frequency (32,768 Hz corresponding to 2^15^ Hz). 

Indeed, the small gap between their prongs makes it difficult for a laser beam to pass through. It is even more critical with mid-infrared (MIR) lasers presenting larger, diffraction-limited, beam waists, like quantum cascade lasers (QCL) for instance. This spectral band, ranging from 3 to 50 μm, is nevertheless of high interest for gas sensing, as it is called the “molecular fingerprint region” for many targeted gases. To circumvent this limitation, one can resort to hollow core fibers to facilitate QCL beam alignment between the QTF’s prongs, leading to a more complex optical setup [4].

A high resonance frequency will make measurements less sensitive, if not impossible, for gases presenting slow vibrational-translational times, such as CO_2_ in the near infrared or MIR. For example, at 2 μm, CO_2_ exhibits a relaxation time of a few microseconds [5], leading to a signal reduction of 60% for a 32,768 Hz tuning fork.

In addition to these drawbacks, standard QTFs present low-quality factors at atmospheric pressure (*Q* ~10,000) compared with their quality factor under vacuum (*Q* ~100,000). As the *SNR* scales as the root mean square of the QTF’s quality factor, it is possible to enhance the sensor sensitivity by improving the detector’s quality factor.

Nonetheless, the use of classical watch QTFs allowed to reach a *NNEA* of 3.3 × 10^−9^ W.cm^−1^.Hz^−1/2^ for C_2_H_2_ at atmospheric pressure, with the use of adapted longitudinal acoustic resonators [6]. Similar values have been reported in almost all of the recent papers on the topic [6,7], even if the best value of 3.75 × 10^−11^ W.cm^−1^.Hz^−1/2^ was reported in 2016 [8]. In comparison, classical PAS sensitivity with acoustical resonators and microphones demonstrates similar sensitivities with a best reported *NNEA* of 2.4 × 10^−10^ W.cm^−1^.Hz^−1/2^ [9].

### 1.2. Custom Quartz Tuning Forks

In order to mitigate these drawbacks, a few custom QTFs have been reported in the literature [10,11,12], presenting T-shape prongs, a larger gap between the prongs from 0.4 to 1 mm, reduced resonance frequency down to 2.8 kHz, and enhanced quality factor in air at atmospheric pressure of 15,000 at best. These resonators were demonstrated to work at the first overtone frequency and making possible dual gas sensing by exciting both fundamental and overtone flexural modes [13]. Another choice is to reduce the gap between the prongs to benefit more from the acoustic pressure actuating the QTF [14], despite the induced elevation of the background noise due to higher residual illumination of the QTF’s prongs. This approach could be considered in the MIR thanks to the use of background noise suppression techniques such as the electrical modulation cancellation method [15] or phase quadrature measurements, which we recently introduced [16]. It is also possible to use different configurations such as off-beam QEPAS to avoid illumination of the QTF’s prongs by the laser [17,18].

### 1.3. Our Custom Quartz Tuning Fork

**Figure 1:**
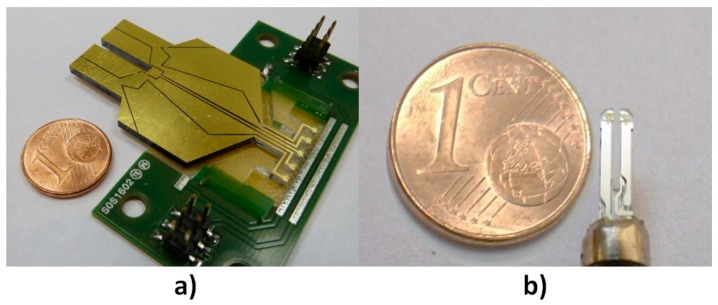
(**a**) Picture of the custom quartz tuning fork used for our experiment. The quartz tuning fork (QTF) presents a 2 mm gap between the prongs. (**b**) Picture of a commercial fork.

In previous works [19,20], we introduced our own custom QTF (Figure 1) with adapted thin film gold electrodes, presenting a gap between the prongs of 2 mm and a frequency of 21,500 Hz. The QTF is 2 mm thick, and its prongs are 13.6 mm high and 8 mm large.

This QTF, called C2, was optimized by the use of an analytical model [20] and through finite element modeling (FEM) using OOFELIE::Multiphysics© [21]. The QTF frequency was a result of the optimization of the quality factor and is still high for photoacoustics. However, we already presented another QTF presenting a lower frequency of 5 kHz [22], allowing us to measure gases with slow relaxation times. C2 presents a quality factor of 450,000 under vacuum and 7600 in air at atmospheric pressure [19], which is very close to the predicted value of 7320, as shown in Table 1. As explained hereafter in detail, this tuning fork was designed to be placed within a specific acoustic cavity, leading to this moderate *Q* factor for the bare QTF.

The total quality factor predicted by our analytical model accounts for anchor losses *Q_support_*, thermoelastic damping of the material *Q_thermo_*, squeezing *Q_squeeze_* due to acoustic waves confinement between structures, viscous losses *Q_viscous_* (both lateral and frontal) due to dissipation into the fluid, and acoustic radiation *Q_acoustic_*. In contrast with classical QTFs, C2 introduces an important energy loss through acoustic radiation. This loss can be expressed as follows through its associated quality factor [20]:

(2) $$Q_{acoustic}=\frac{64}{3}\;\frac{\rho_{q}}{\rho_{f}}\frac{1}{k_{v}^{4}el{\left(e+g\right)}^{2}},\;$$

where $\rho_{q}$ is the quartz volumic density, $\rho_{f}$ is the fluid volumic density, *k_v_* is the acoustic wave vector, *e* is the QTF branch width, *g* is the gap between its prongs, and *l* is the QTF thickness.

To recover this radiated energy, an enclosing cavity was added to our QTF to confine the acoustic waves emitted by the QTF itself [19], providing an unmatched quality factor of 45,400 in air, around 21,500 Hz, along with a better acoustic isolation from the environment. This value is lower than the theoretical *Q* factor calculated in Table 1 because of remaining acoustic losses that can be estimated to be *Q_acoustic_* = 75,000, coming from the complexity to recover the acoustic waves with a perfect phase-match. To our best knowledge, it is the first time such a cavity has been used to recover the acoustic waves emitted by the QTF, in photoacoustic spectroscopy. 

**Figure 2:**
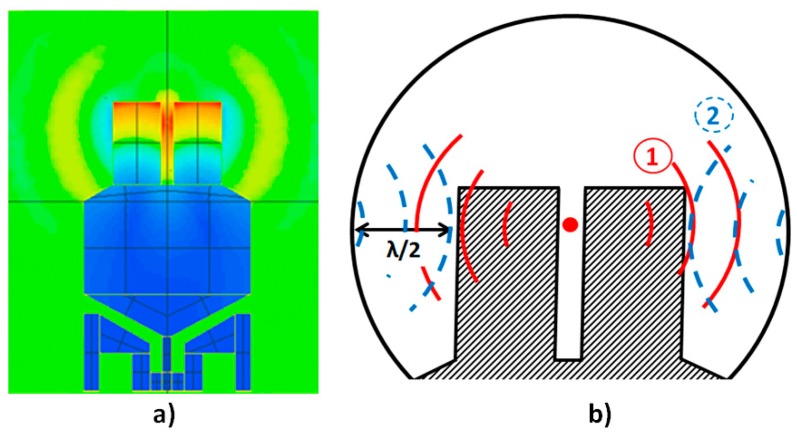
(**a**) Acoustic radiation of our QTF simulated by finite element modeling (FEM). The QTF was excited by applying a pressure force on the QTF prongs. (**b**) Principle scheme of acoustic recovery—firstly, emission of acoustic waves by the QTF; and secondly, reflection thanks to the acoustic recovery device.

FEM simulations of our system demonstrated that the radiating acoustic pressure field was remarkably cylindrical and in the QTF’s plane, as shown in Figure 2. The cavity was thus designed as a cylinder whose radius was adapted through FEM in order to generate acoustic stationary waves in phase with the QTF’s emitted acoustic waves. The distance between the prongs and the cavity wall is approximately equal to half the acoustic wavelength *λ*.

**Figure 3:**
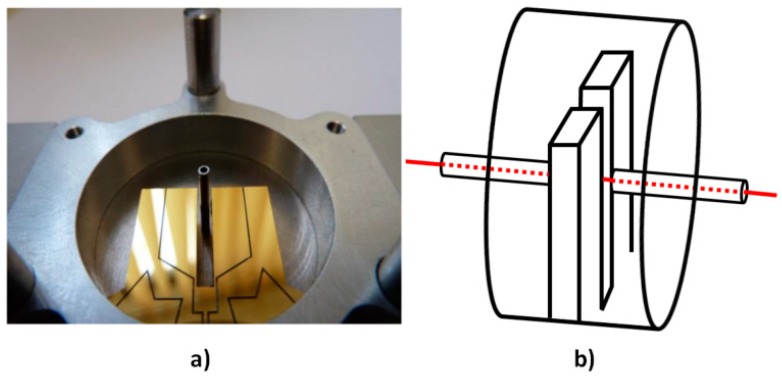
(**a**) Picture of the QTF within its acoustic cavity: the microresonators for acoustic amplification and the surrounding cylinder for acoustic recovery. (**b**) Schematic of our QTF used with its acoustic recovery cavity and acoustic microresonators, with a laser beam passing through (in red).

Finally, acoustic microresonators have been added to our system (Figure 3). This configuration called on-beam [6] allows enhancing the sensor sensitivity by increasing the acoustic pressure near the QTF. Usually two longitudinal acoustic resonators are added to the QTF excited in its fundamental mode providing an increase of 15 to 40 [6,23,24] on the sensor sensitivity. Unfortunately, this comes along a great diminution of the overall quality factor, which is difficult to predict analytically [25].

Concerning the electronics, the used circuit is an analog circuit including one transimpedance amplifier, followed by a voltage amplification stage made of two band-pass filters. Measurements of the sensor’s noise spectrum and results from a simulation software confirmed that the noise at resonance is given by the Brownian movement of particles. In other words, the sensor noise is limited by its fundamental limit. 

In the following paragraphs, we experimentally study the influence of the addition of the previously introduced acoustic devices.

## 2. Influence of Added Acoustic Devices

### 2.1. Environment Isolation by an Acoustic Cavity

As presented previously, we developed a cavity allowing acoustic recuperation of the acoustic waves emitted by our custom quartz tuning fork (Figure 3). One cavity was designed by FEM—it is a stainless steel cylinder with a 16 mm radius and a 16 mm height. 

In order to study the isolation of our system to ambient acoustic noise, we placed it in an isolated chamber together with a loud speaker generating a pure sine wave. Without moving the loud speaker, we changed the configuration of our system and measured its response when varying the frequency of the generated sound. Despite the chamber not being anechoic, we expect this experiment to provide representative data on our system, as we do not expect the chamber to have a huge influence on our detector. 

**Figure 4:**
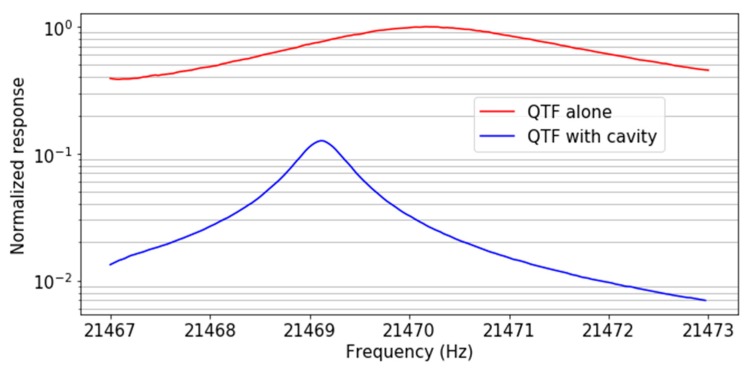
Normalized sensitivity to the external sound, for the bare C2 QTF (red) and for C2 with its acoustic recovery cavity without microresonators (blue).

In Figure 4, we trace the frequency responses of each configuration after normalizing them by the response of the configuration “QTF alone”.

We also measured the *Q* factor and resonance frequency with an impedance-meter (4294A, Agilent Technologies, Santa Clara, CA, USA), and obtained the results presented in Table 2.

The cavity is promising for the improvement of the system’s acoustic isolation. Indeed, a reduction of about eight times of the acoustic signal at the QTF’s resonance frequency is observed, along with a quality factor six times better compared with the case in which no cavity is employed. This isolation can be partly explained by the enhancement of the system’s overall quality factor, and may also be explained by the acoustic shielding provided by the walls of the acoustic cavity.

### 2.2. Design Optimization of the Microresonators

After having characterized the effect of the cavity on the QTF, we study the influence of the geometry of the microresonators on the quality factor when using such a cavity. These longitudinal acoustic resonators are placed on both sides of the QTF’s prongs. Their introduction modifies the global quality factor in a non-trivial way [25].

In theory, the optimum tube length would be equal to *λ*/2 if the tubes were decoupled from each other and from the QTF and equal to *λ*/4 if the tubes were totally coupled. In practice, the optimum tube length is somewhere between these two values [6], and has to be determined from FEM simulations or, better, experimentally. In addition, these microresonators should always be chosen with the smallest possible diameter because the acoustic pressure scales inversely with the diameter. On the other hand, the diameter must be large enough for the laser beam to pass through. 

**Figure 5:**
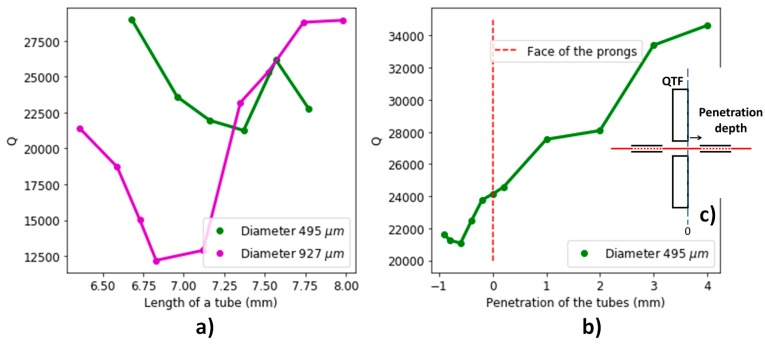
(**a**) Quality factor of the system (QTF + microresonators) as a function of the length of the tubes, for two different diameters of the tubes and a penetration depth of the tubes of −0.8 mm with respect to the face of the prongs. (**b**) Same as (**a**) for tubes of same length and diameter, depending on the depth of penetration of the tubes within the gap between the QTF’s prongs. The quality factor is measured with an impedance-meter. (**c**) Schematic of the penetration depth.

Figure 5 shows the system quality factor over inner diameter, length, and penetration depth of the microresonators.

When varying the length of the microresonators, a quality factor minimum is identified, corresponding to a maximum acoustic coupling with the QTF. A better coupling comes along with a better acoustic pressure amplification, and thus a better signal-to-noise ratio [6]. In Figure 5b, we see that the *Q* factor decreases when reducing the distance to the prongs, except between −0.8 to −0.5 mm, where it increases. The reason for this is a reduced coupling between the resonators when the gap between the needles becomes smaller.

We can conclude that the optimal microresonators should be 7.3 mm long for an internal diameter of 495 μm and a penetration depth of −0.8 mm. Adding the microresonators to the QTF and acoustic recovery cavity reduces the quality factor to half its initial value. The resulting *Q* factor of 21,000 is ten times the quality factor obtained for watch QTFs with adapted microresonators [6].

Despite the drop on the quality factor, the addition of such resonators allowed a sensitivity enhancement of about 10, as we will detail hereafter.

## 3. Gas Detection

### 3.1. Targeted Absorption Line

**Figure 6:**
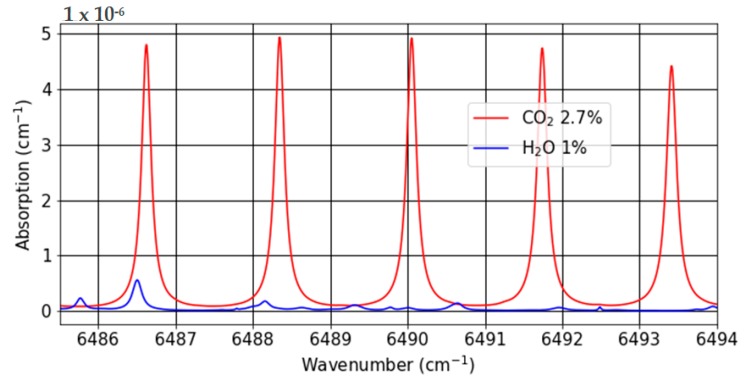
Calculated absorption spectra of CO_2_ (red) and H_2_O (blue) at atmospheric conditions (*P* = 101,325 Pa, *T* = 300K) using the HITRAN database.

To measure the sensitivity of our system, we use a certified CO_2_ mixture of 2.7% in nitrogen. The laser used for the detection is a standard telecom laser diode emitting around 1.5 μm. We target the 6490.05 cm^−1^ absorption line of CO_2_ with an absorbance of 5 × 10^−6^ cm^−1^ for a 2.7% concentration and a full width at half maximum (FWHM) of 0.16 cm^−1^. The ambient humidity is monitored and corresponds to typical atmospheric humidity (~10,000 ppm), even though water vapor interferes very weakly with our measurement (Figure 6).

### 3.2. Setup

**Figure 7:**
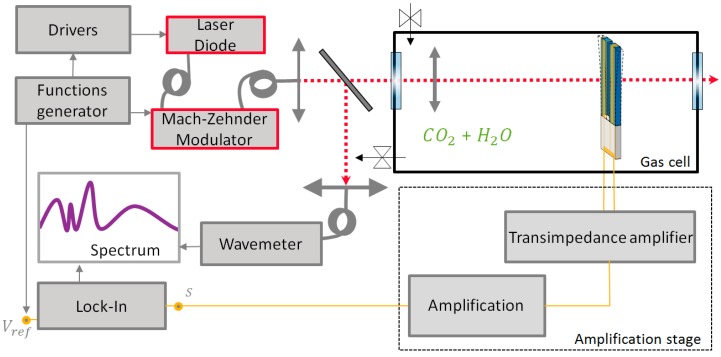
Detection scheme for quartz enhanced photoacoustic spectroscopy (QEPAS) CO_2_ detection.

We use a wavelength modulation scheme with second harmonic detection. The setup is presented in Figure 7. The laser diode temperature and current are controlled (ITC 510, Thorlabs, Newton, MA, USA) and the laser wavelength is modulated with a *f*_0_/2 sinusoid wave (33000B, Agilent Technologies, Santa Clara, CA, USA), where *f*_0_ is the QTF’s fundamental vibration frequency. The wavelength is measured continuously with a wavemeter (WS6 785, HighFinesse, Tübingen, Germany) to keep a good match between the absorption line and the laser optical frequency. The QTF’s signal is first amplified by means of a low noise transimpedance amplifier, and sent to a lock-in amplifier (SR530, Stanford Research Systems, Sunnyvale, CA, USA) with a 1/16 Hz bandwidth, measuring the second harmonic signal.

The cell contains two wedged windows with an antireflection coating, a lens of focal length 200 mm, the tuning fork, and its transimpedance amplifier. The cell is filled with the gas mixture before each measurement. The custom QTF C2 is surrounded by a 16 mm radius stainless steel cylinder acting as an acoustic cavity allowing in-phase recovery of the emitted acoustic waves, as explained in Section 1.3. Two longitudinal acoustic resonators, which are cut from stainless steel hypodermic needles, are placed along the laser beam, inside the cavity. For these needles, various parameters have been tested: internal diameter, length, position with respect to the QTF stem, and penetration depth within the gap between the prongs, as explained in Section 2.2. We also designed a monolithic cavity including the needles in bulk stainless steel to ease the optical alignment.

### 3.3. CO_2_ Detection

After having optimized the design of the acoustic microresonators, we performed the detection of CO_2_ at 2.7% in nitrogen. The average modulated laser power was measured in front of the QTF, to be *P* = 17 mW.

By measuring the signal amplitude and the RMS noise within the detection bandwidth, we find a *NNEA* of 3.3 × 10^−8^ W.cm^−1^.Hz^−1/2^ for our QTF alone and we reach a sensitivity of 3.7 × 10^−9^ W.cm^−1^.Hz^−1/2^ for the QTF with the acoustic cavity and the best microresonators cut from needles, providing an enhancement of a factor 10. This enhancement is slightly lower than reported values usually ranging from 15 to 40 [6,23,24] for on-beam QEPAS using dual tube resonators exciting the QTF’s fundamental mode. This might come from the enhanced acoustic coupling between the QTF and the microresonators, as the QTF emits acoustic waves disturbing the microresonators’ acoustic resonance.

We also designed the acoustic cavity in a bulk piece of stainless steel with integrated tubes of different sizes, allowing good alignment repeatability. With machined tubes of diameter 0.7 mm and length 7.3 mm, we obtained a *NNEA* of 7.6 × 10^−9^ W.cm^−1^.Hz^−1/2^. The monolithic acoustic cavity could not be machined with microresonators of diameter less than 0.7 mm, because it becomes difficult for suppliers to guarantee the tolerances and a good inner surface polishing. Nonetheless, it would be interesting to carry on in this direction to verify that we can obtain the same results than those obtained with the hypodermic needles.

**Figure 8:**
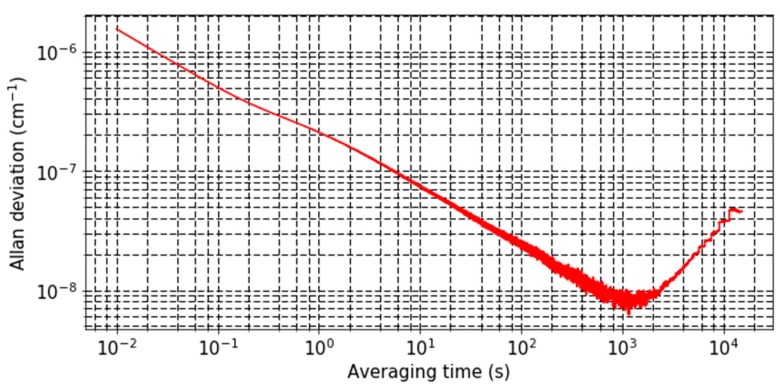
Allan deviation of the measured absorption of 2.7% of CO_2_ at 6490.05 cm^−1^.

Finally, an Allan deviation measurement was performed (Figure 8) to analyze the sensor’s stability. The C2 QTF was used with both acoustic devices (acoustic recovery and optimal microresonators) to measure the photoacoustic signal in 2.7% of CO_2_ at normal conditions of pressure and temperature. 

We can divide the analysis of the curve in three parts. From 10 ms to 1 s, we see an averaging surely owing to acoustic phenomena within the acoustic devices, from 1 s to 1150 s the averaging of the thermal noise of the QTF and beyond, we see that the sensor is drifting due to temperature induced frequency shift. Compared with other QEPAS measurements [26,27], we see that the drift occurs later—it could be explained by the fact that our QTF is much larger than any other QTF, making it less sensitive to drifts. A minimum deviation of 9 × 10^−9^ cm^−1^ is obtained for a 19 min averaging time, corresponding to 44 ppmv of CO_2_ detected at 1.54 μm. This concentration value could easily be enhanced targeting a stronger absorption line of CO_2_ at MIR wavelengths (QCLs) and by raising the laser power. 

## 4. Conclusions

A quartz-enhanced photoacoustic gas detector with a custom quartz tuning fork was demonstrated. Its lower detection frequency makes it more suitable to address gases with slower relaxation times. Moreover, owing to the wider gap between the QTF’s prongs (2 mm) than for standard QTFs, a better sensitivity and less experimental difficulties are expected with the use of larger laser beams in the mid-infrared (typically from QCLs). 

To improve the performance, the custom QTF is enclosed in a cylindrical acoustic cavity with longitudinal acoustic microresonators. The acoustic cavity enables the recovery of the energy radiated though generated acoustic waves, while the acoustic microresonators provide an additional enhancement of the sensitivity through an increase of the pressure between the QTF’s prongs. We designed and optimized the acoustic cavity and the acoustic resonators, showing the influence of different parameters of these objects. The best geometry for the tubes was found, with 7.3 mm length, 0.5 mm diameter, and penetration depth of −0.8 mm inside the QTF’s prongs when using a laser diode at telecom wavelength. We also designed bulk cavities with larger needles diameters and optimal lengths to accommodate for larger laser beams as those emitted by MIR lasers.

We showed that the cavity used for acoustic recovery also allowed efficient acoustic isolation against ambient noise at the QTF frequency. Finally, we performed an Allan deviation showing an absorption detection limit of 9 × 10^−9^ cm^−1^ for CO_2_ detection around 1.5 μm.

Future work will include the optimization of the acoustic cavity and adaptation of the design for use with longwave infrared quantum cascade lasers.

## Figures and Tables

**Table 1:** Theoretical performances of our custom quartz tuning fork (QTF) [19].

	Bare QTF	QTF + Acoustic Recovery
Frequency	21.23 kHz	21.23 kHz
*Q_support_*	1 × 10^6^	1 × 10^6^
*Q_thermo_*	5.48 × 10^7^	5.48 × 10^7^
*Q_squeeze_*	3.82 × 10^7^	3.82 × 10^7^
*Q_viscous_* (lateral)	2.91 × 10^5^	2.91 × 10^5^
*Q_viscous_* (frontal)	2.96 × 10^5^	2.96 × 10^5^
*Q_acoustic_*	7.76 × 10^3^	-
***Q total***	**7.32 × 10^3^**	**1.27 × 10^5^**

**Table 2:** Acoustic isolation granted by our cavity compared with the case in which no cavity is employed.

	C2 Alone	C2 with Cavity
Frequency (Hz)	21,470.22	21,469.12
*Q* factor	7600	45,441
Normalized sensitivity to external sound	1	1/8
